# Changes in Epidemiological Characteristics of Varicella and Breakthrough Cases in Ningbo, China, From 2010 to 2023: Surveillance Study

**DOI:** 10.2196/71691

**Published:** 2025-06-18

**Authors:** Xingqiang Pan, Yan Zhang, Xuefei Zhao, Dandan Zhang

**Affiliations:** 1 Ningbo Center for Disease Control and Prevention, No. 1166 Fanjiangan Road, Haishu District, Ningbo, 315000, China, 86 057487680154

**Keywords:** varicella, breakthrough case, epidemiology, disease surveillance, infectious, monitoring, vaccine, nonpharmacological interventions, machine learning

## Abstract

**Background:**

Varicella is a prevalent respiratory infectious disease. Continuous monitoring is essential to understand evolving epidemiological patterns, particularly given the impact of vaccination and recent nonpharmacological interventions.

**Objective:**

This study aims to monitor the epidemiological characteristics of varicella and the changes in breakthrough cases to inform adjustments in immunization strategies and enhance prevention efforts.

**Methods:**

From 2010 to 2023, varicella incidence was monitored using active (2010-2011) and passive (2012-2023) surveillance methods. Data were obtained from the Chinese Center for Disease Prevention and Control’s information system and Ningbo’s Immunization Information System. The study period was divided into four intervals to analyze trends. A birth cohort (2009-2013) was established to examine breakthrough cases. A recurrent neural network model was constructed for deep learning analysis of incidence trends and the impact of nonpharmaceutical interventions.

**Results:**

Between 2010 and 2023, a total of 70,163 varicella cases were reported in Ningbo. Seasonal distribution indicated two incidence troughs before 2020 and only one from 2020 to 2023. The predominant age of onset was 10‐14 years, accounting for 23.93% (16,795/70,163) of cases. From 2010 to 2013, the highest incidence was among children aged 5‐9 years; from 2014 to 2019, it shifted to those aged 10‐14 years; and from 2020 to 2023, it was primarily among individuals aged 15‐19 years. Following the introduction of a second vaccine dose (2014‐2019), incidence among children younger than 10 years of age decreased, notably by 59.54% in those aged 1‐4 years. Conversely, incidence among individuals aged 10 years and older increased, particularly by 123.78% in the 15‐19 years age group, with a significant upward trend (*P*_trend_<.001). From 2020 to 2023, although incidence rates increased across age groups 15 years and older, the rise was modest. The average annual incidence rate of breakthrough cases after one vaccine dose was 83.40/100,000 (range, 51.21‐119.50/100,000), significantly higher than the 24.80/100,000 (range, 17.67‐32.90/100,000) observed after two doses. However, the incidence of breakthrough cases after the first dose declined following the implementation of the 2-dose program. The median time from vaccination to breakthrough case occurrence was 27 (IQR 17.50‐48) months. The recurrent neural network model demonstrated high accuracy (mean squared error, 49.96) and indicated that implementation of emergency response and community lockdown measures in early 2020 correlated with a divergence between predicted and actual case numbers, suggesting an impact of nonpharmaceutical interventions on varicella transmission.

**Conclusions:**

The significant shifts in varicella epidemiology between 2010 and 2023 highlight the importance of continuous monitoring and proactive immunization adjustments. We recommend enhanced varicella surveillance focusing on adult populations, and a targeted increase in 2-dose vaccine coverage, particularly in high-risk environments such as high schools and universities.

## Introduction

Varicella, a highly contagious disease caused by the varicella-zoster virus, is primarily transmitted through direct contact with the rash of varicella or herpes zoster, as well as by inhaling aerosolized droplets from the respiratory secretions of patients with varicella [1]. While varicella is typically mild and self-limiting in healthy children, it can be life-threatening for immunosuppressed patients, pregnant women, and older people. In high-risk groups, varicella infection may result in severe complications such as pneumonia, encephalitis, hepatitis, organ failure, and acute cerebellar ataxia [2]. According to the World Health Organization, the annual global burden of varicella is estimated at around 140 million cases, leading to 4,200,000 harsh complications necessitating hospitalization, and resulting in 4200 deaths [3]. Notably, China reported a total of 3,047,715 varicella cases between 2016 and 2019, with an average annual incidence rate of 55.05 per 100,000 [4]. The incidence rate significantly increased from 35.50 per 100,000 in 2016 to 70.14 per 100,000 in 2019, surpassing other legally notifiable infectious diseases like measles and rubella. Since varicella is not a notifiable infectious disease in China, the actual incidence rate is probably higher than the reported rate. This underscores the pressing need for effective prevention strategies to tackle the significant public health impact of varicella infection.

The introduction of the varicella vaccine has been crucial in disease control, significantly reducing the incidence, hospitalizations, and mortality rates associated with varicella [5,6]. The vaccine was first licensed for use in the United States in 1995. In 2006, a 2-dose schedule was recommended for children, with the first dose given at 12‐15 months and the second dose between 4 and 6 years of age [6]. This immunization strategy has also been adopted by Germany, Australia, and Canada. In contrast, China introduced the varicella vaccine in 1998 but did not include it in the national immunization program, opting for a single-dose strategy targeting children aged 12‐18 months. Starting in 2014, Ningbo implemented a 2-dose vaccination strategy, with the first dose administered at 12‐15 months and the second dose at age 3 years and older. Vaccination in Ningbo is voluntary and self-funded, achieving a vaccination rate exceeding 90% for one dose and over 70% for two doses [7,8]. The use of the varicella vaccine in children has led to changes in the epidemiological characteristics of varicella, including a shift toward older age groups [3]. Nonpharmaceutical preventive measures for COVID-19 may also impact the epidemiological features of varicella. Studies indicated that the effectiveness of a single varicella vaccine dose diminishes over time, contrasting with the sustained effectiveness of the 2-dose vaccine [9]. Even if vaccinated, breakthrough cases of varicella can still occur. Although usually less severe, these cases can contribute to the spread of varicella within a community.

The World Health Organization emphasizes the need for thorough varicella surveillance to understand the disease’s spread and to guide public health efforts in prevention and control. Since 2009, targeted surveillance efforts have been implemented, establishing specific varicella surveillance points in Ningbo, China, to monitor disease incidence and spread. In 2012, the Ningbo Municipal Bureau of Health required all health care facilities to report varicella cases to the China Information System for Disease Control and Prevention (CISDCP) within 24 hours, and this measure enhanced the accuracy and promptness of data collection. This study aims to use both active and passive surveillance data to analyze the epidemiological features of varicella and breakthrough cases.

## Methods

### Setting

Ningbo, a subprovincial city in Zhejiang province, East China, is one of the five state-planning cities in the country. Before 2016, Ningbo comprised 6 urban districts (Haishu, Jiangbei, Jiangdong, Zhenhai, Beilun, and Yinzhou), 3 satellite county-level cities (Fenghua, Yuyao, and Cixi), and 2 rural counties (Ninghai and Xiangshan). Following a regional restructuring in 2016, Ningbo now includes 6 urban districts: Haishu, Jiangbei, Zhenhai, Beilun, Yinzhou, and Fenghua; 2 county-level cities: Yuyao and Cixi; and 2 counties: Ninghai and Xiangshan. The city spans 9816.2 km² of land and 8355.8 km² of sea. As of the end of 2022, Ningbo had a population of 9.618 million permanent residents. Since 2005, the Ningbo Center for Disease Control and Prevention has operated the Ningbo Immunization Information System, a computerized system that maintains immunization data and demographic information on vaccines for the Ningbo region [10].

### Active Monitoring Only, 2010-2011

The varicella active surveillance project [10] was conducted in Yuyao City, Yinzhou District, and Ninghai County during 2010 and 2011, through a collaboration between the Yuyao Center for Disease Prevention and Control (CDC), Yinzhou CDC, Ninghai CDC, and Ningbo CDC. Yuyao City is located in the western part of Ningbo, on the southern bank of the Yangtze River Delta. Yinzhou is a central district of Ningbo, covering an area of 1380 square kilometers. Ninghai County is situated to the south of Ningbo and spans approximately 1931 square kilometers. The populations of Yuyao, Yinzhou, and Ninghai County were 830,000, 730,000, and 580,000, respectively.

Since mid-2009, local health providers and physicians in the varicella surveillance areas have been required to electronically report any diagnosed varicella case to CISDCP within 24 hours. The epidemiologic information recorded for each case includes age, gender, current home address, household registration, date of symptoms onset, date of diagnosis, vaccine history, and other relevant details. Public health doctors in the surveillance area were also responsible for actively identifying varicella cases in hospitals, community health service centers, schools, and kindergartens on a weekly basis. If an unreported varicella case was found, public health doctors would report it through CISDCP.

### Passive Surveillance, 2012-2023

Since 2012, under the requirement of the health administration department in Ningbo City, all medical institutions in the city have been required to report varicella cases through CISDCP. The CDC conducted quarterly investigations on underreporting to improve the quality of reporting.

#### Varicella Vaccine Immunization Strategy Adjustment in 2014

Ningbo City implemented a 2-dose varicella immunization strategy. Children receive voluntary and self-paid varicella vaccination. The recommended timing for the first dose is at 1 year of age, and the recommended timing for the second dose is between 3 and 4 years of age.

#### Nonpharmaceutical Intervention Measures

Starting from 2020, various nonpharmaceutical intervention (NPI) measures have been implemented by the government to control the COVID-19 epidemic. These measures include maintaining social distancing, wearing masks, school closures, and community lockdowns, which have had a significant impact on the transmission of other respiratory infectious diseases.

### Diagnosis of Varicella and Breakthrough Cases

Varicella diagnostic criteria [11] are characterized by typical clinical manifestations, namely the presence of characteristic rashes that progress from macules to papules and then to vesicles before crusting. It is usually accompanied by fever and systemic symptoms such as malaise. If there are no typical clinical manifestations, the diagnosis needs to be made in conjunction with epidemiological history. The incidence of varicella in Ningbo is determined by dividing the number of reported cases by the corresponding population size. Population data for each age group is sourced from the Ningbo Municipal Public Security Bureau.

A birth cohort was established for children born between 2009 and 2013 who received one dose of varicella vaccine at the surveillance sites. Their varicella incidence was monitored through CISDCP. Varicella vaccine administration information was obtained through Ningbo’s Immunization Information System.

Breakthrough varicella is defined as the occurrence of clinically diagnosed varicella in individuals who present with characteristic symptoms or have a documented epidemiological exposure history (as previously described), with symptom onset occurring more than 42 days after vaccination [12]. Furthermore, the rate of breakthrough infections has been delineated as the ratio of breakthrough infections occurring in children who have undergone varicella vaccination.

### Data Extraction and Analysis

The varicella cases recorded between the years 2010 and 2023 were sourced from the comprehensive infectious disease surveillance and CISDCP. According to the temporal onset of illness, the cases were stratified into four phases for the purposes of descriptive analysis: 2010‐2011, 2012‐2013, 2014‐2019, and 2020‐2023. In the context of regional distribution analysis, and owing to specific regional modifications in Ningbo, the interval from 2014 to 2019 was further subdivided into two distinct phases: 2014‐2015 and 2016‐2019. Chi-square statistical tests were used to examine the variations in age and gender across the different phases, while trend chi-square tests were used to assess the evolving trends of various age cohorts within each phase.

To evaluate the impact of NPIs on the incidence of chickenpox, we developed an advanced deep-learning framework using recurrent neural networks. The model was trained on a dataset of chickenpox cases recorded between January 1, 2012, and December 31, 2018, and validated using data from January 1, 2019, to December 31, 2019. Predictions generated by the model were compared with actual incidence data from January 1 to June 30, corresponding to the implementation of Level 1 Emergency Response on January 22 and community lockdown measures introduced on February 2. The training process used a sliding window technique to forecast future case numbers, and model performance on the validation dataset was evaluated using metrics such as mean squared error, root mean squared error, and mean absolute error. The final model was then used to predict chickenpox incidence for the period from January 1, 2020, to June 30, 2020. These predictions were analyzed to assess the influence of NPIs by comparing the forecasted incidence with the observed data for the same timeframe.

The Python programming language facilitated the processes of data cleansing, analytical procedures, and data visualization. The 2-sided *P* values were documented, adhering to a significance threshold of *P*<.05. All analytical procedures were conducted using Python software (version 3.10; Python Software Foundation) [13].

### Ethical Considerations

This study used secondary surveillance data on varicella cases obtained from the CISDCP. The study protocol was reviewed and approved by the Biological and Medical Ethics Committee of Ningbo Center for Disease Control and Prevention (approval: 202312), ensuring adherence to ethical guidelines for public health research. The original data collection involved informed consent procedures as per national regulations; the Ningbo CDC Ethics Committee specifically granted approval for this secondary analysis without requiring additional consent from individual patients. All data were fully anonymized and deidentified prior to access and analysis, protecting patient privacy and confidentiality. No personal identifiers were present in the dataset provided to the researchers. No compensation was provided to participants as this study involved the analysis of routinely collected surveillance data. The manuscript and supplementary materials do not contain any images of individual participants. We confirm that all data handling and analysis were conducted in compliance with relevant ethical standards and regulations for public health surveillance.

## Results

### Pathogenesis Overview

Between 2010 and 2023, a total of 70,163 varicella cases were documented in Ningbo. Within the designated monitoring region, 4753 cases were reported during 2010‐2011. In the birth cohort from 2009 to 2013, there were 1404 varicella cases, including 912 classified as breakthrough cases. The overall incidence rate for breakthrough cases was 47,149 per 100,000 individuals. The frequency of breakthrough cases increased over time postvaccination, peaking in 2022 at 6307 per 100,000. The annual incidence of varicella rose from 63.89 per 100,000 in 2010 to 98.42 per 100,000 in 2013. It remained relatively stable between 2014 and 2017, ranging from 85.58 to 99.60 per 100,000, before increasing to 121.75 per 100,000 in 2018. A significant decline began in 2020, with the incidence rate dropping from 71.79 per 100,000 to 6389 per 100,000 (Table 1).

**Table 1.** The prevalence of varicella in Ningbo, 2010‐2023

Year	Number of cases	Incidence rates (1/100,000)	
		Incidence of rate	BVR^a^ of the birth cohorts
2010	2689	43.63	0.58
2011	3568	57.89	10.52
2012	4949	83.32	24.55
2013	5646	96.48	35.07
2014	5676	90.05	44.42
2015	5698	98.42	32.73
2016	5039	85.59	23.97
2017	5748	99.61	24.55
2018	7026	121.76	35.66
2019	6861	118.90	29.23
2020	4307	71.79	22.80
2021	4759	79.32	30.39
2022	4265	69.30	30.39
2023	3932	63.89	33.32

a BVR: breakthrough varicella incidence rate.

### Spatial Distribution

**Figure 1.**
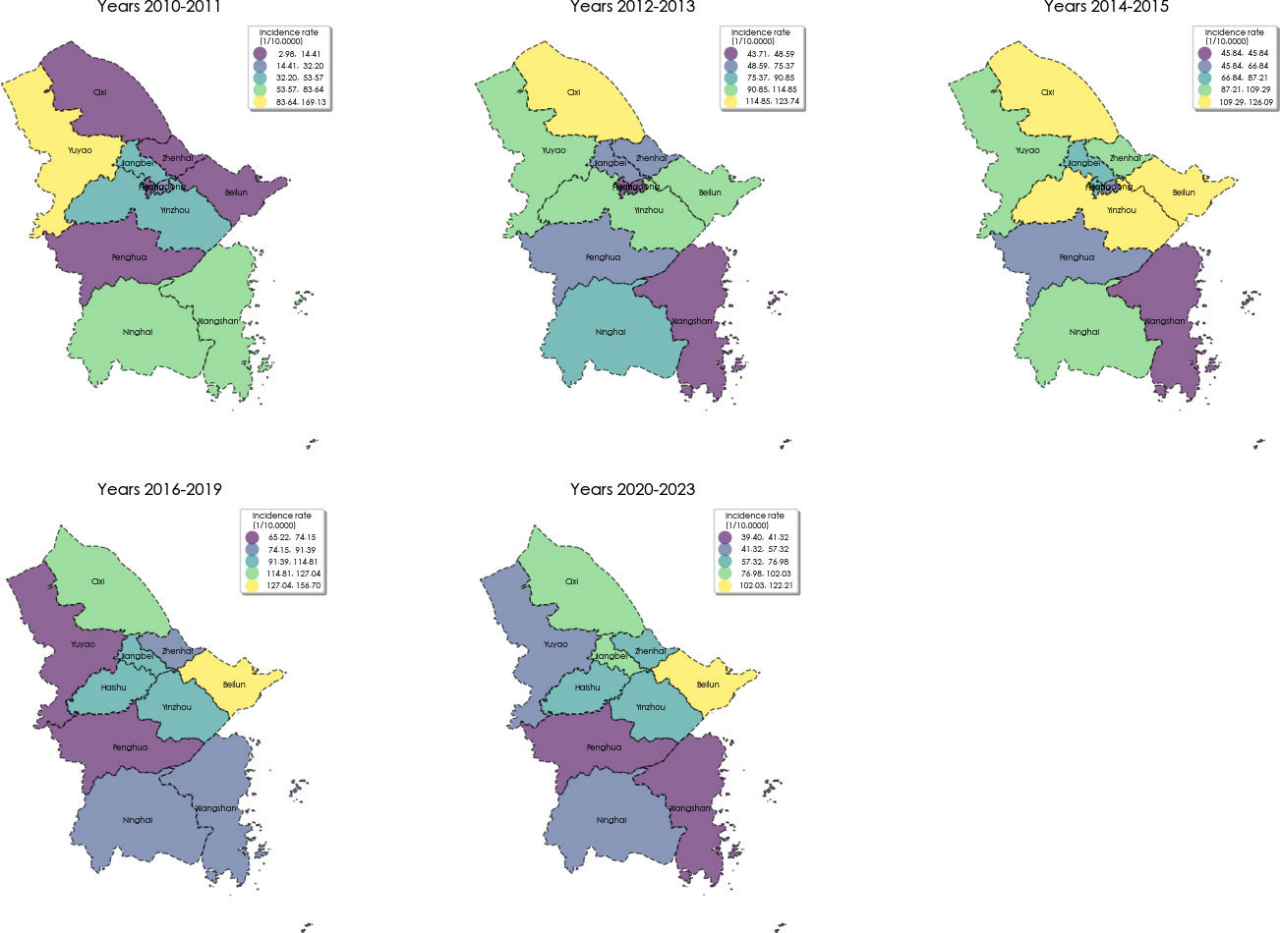
The spatial distribution of varicella in Ningbo, 2010‐2023.

Between 2010 and 2023, the municipalities with the highest average annual incidence rates of varicella were Beilun District, Yinzhou District, and Cixi City, with rates of 113.44, 105.95, and 99.70 per 100,000, respectively. Figure 1 illustrates the trajectory of these rates across each county and city district. During the active surveillance period, Yuyao City recorded the highest average annual incidence rate of 169.13 per 100,000 in 2010‐2011. In the subsequent periods of 2012‐2013 and 2014‐2015, Cixi City reported the highest rates at 123.73 and 126.09 per 100,000, respectively. From 2016 to 2019 and 2020 to 2023, Beilun District had the highest rates at 156.70 and 122.21 per 100,000, respectively.

### Temporal Distribution

Between the years 2010 and 2023, there were documented instances of varicella cases on a monthly basis in the city of Ningbo. Table 2 illustrates that during the periods 2010‐2011, 2012‐2013, and 2014‐2019, two significant peaks in cases occurred: from May to July and from October to January of the following year. The incidence rates for May to July were 29.84% (1867/6257), 22.38% (2371/10,595), and 25.27% (9108/36,048), while the rates for October to January were 47.16% (2951/6257), 47.16% (2951/6257), and 45.51% (16,406/36,048), respectively. The months with the lowest incidence rates were February and September, with September accounting for 4.12% (258/6257), 4.95% (524/10,595), and 4.49% (1620/36,048), and February accounting for 2.70% (169/6257), 4.91% (520/10,595), and 5.90% (2127/36,048) across the respective years. From 2020 to 2023, the incidence rates in August and September showed no significant variation, while the number of cases from October to January remained high, comprising 45.32% (7823/17,263) of the total cases.

**Table 2.** Month of varicella onset by four phases in Ningbo.

Month of disease onset	Number of cases				
	Years 2010‐2011	Years 2012‐2013	Years 2014‐2019	Years 2020‐2023	Total
1	614	1072	3061	1252	5999
2	258	524	1620	752	3154
3	299	513	2049	899	3760
4	510	695	2506	1061	4772
5	601	816	3340	1456	6213
6	809	793	2899	1268	5769
7	457	762	2869	1394	5482
8	203	548	2232	1284	4267
9	169	520	2127	1326	4142
10	418	996	3287	1808	6509
11	817	1556	4969	2659	10,001
12	1102	1800	5089	2104	10,095

### Population Distribution

**Figure 2.**
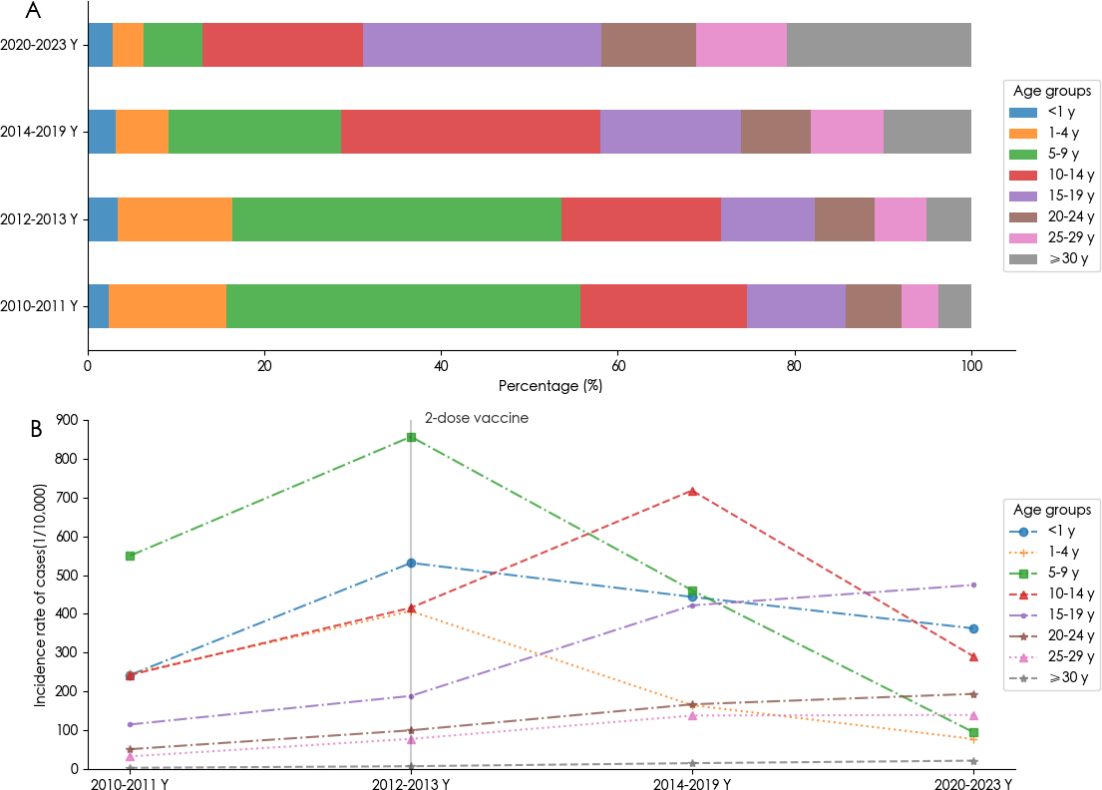
The age distribution of varicella in Ningbo. (A) The composition ratio of age by the phases. (B) The incidence rates by age during the phases.

From 2010 to 2023, the age groups with the highest number of cases were primarily 10‐14, 5‐9, and 15‐19 years, comprising 23.93% (16,795/70,163), 20.86% (14,639/70,163), and 17.42% (12,224/70,163) of the total cases, respectively. Notably, between 2010 and 2011, the 5‐9 age group had the highest proportion, accounting for 40.10% (2509/6257) and 37.31% (3953/10,595) of the total cases, respectively. In contrast, from 2014 to 2019, the 10‐14 age group had the highest proportion, reaching 29.34% (10,577/36,048). From 2020 to 2023, the 15‐19 age group had the highest proportion, accounting for 26.97% (4656/17,263) of the total cases (Figure 2A).

From an analytical perspective on incidence rates, the age groups younger than 1 year and 5‐9 years demonstrated a declining trend from 2012‐2013 to 2020‐2023, with reductions of 31.70% and 80.96%, respectively. In contrast, the 10‐14 year age group experienced a significant increase of 72.68% from 2012‐2013 to 2014‐2019, followed by a decrease of 29.97% by 2020‐2023. The age groups 15‐19 years, 20‐24 years, 25‐29 years, and 30 years and older showed consistent upward trends from 2010‐2011 to 2020‐2023, with increases of 312.99%, 278.79%, 327.99%, and 575.62%, respectively. These trends were statistically significant, as determined by the trend chi-square test (all *P*<.001; and Table 3) (Figure 2B).

**Table 3.** The incidence rates of varicella by age and gender in Ningbo.

Characteristics	Years 2010‐2011	Years 2012‐2013	Years 2014‐2019	Years 2020-2023	Difference between years 2010‐2011 and 2020‐2023		Difference between years 2012‐2013 and 2014‐2019		Difference between years 2012‐2013 and 2020-2023		Difference between years 2014‐2019 and 2020-2023	
					%	*P* value	%	*P* value	%	*P* value	%	*P* value
Gender												
Male	61.86	101.79	109.15	76.31	64.56	<.001	7.23	<.001	–25.04	<.001	–30.09	<.001
Female	46.60	81.27	91.30	62.76	74.40	<.001	12.34	<.001	–22.77	<.001	–31.26	<.001
Age groups (years)												
<1	242.39	531.46	443.73	362.81	49.68	<.001	–16.51	<.001	–31.73	<.001	–18.24	<.001
1‐4	245.95	407.03	164.68	77.50	–68.49	<.001	–59.54	<.001	–80.96	<.001	–52.94	<.001
5‐9	549.74	856.84	460.81	95.44	–82.64	<.001	–46.22	<.001	–88.86	<.001	–79.29	<.001
10‐14	242.44	415.85	718.10	291.22	20.12	<.001	72.68	<.001	–29.97	<.001	–59.45	<.001
15‐19^a^	114.91	188.53	421.90	474.57	312.99	<.001	123.78	<.001	151.72	<.001	12.48	<.001
20‐24^a^	51.16	99.83	166.79	193.79	278.79	<.001	67.07	<.001	94.12	<.001	16.19	<.001
25‐29^a^	32.58	77.82	137.91	139.44	327.99	<.001	77.22	<.001	79.18	<.001	1.11	<.001
≥30^a^	3.20	7.32	15.16	21.62	575.62	<.001	107.10	<.001	195.36	<.001	42.61	<.001
Total	50.76	90.53	104.11	70.13	78.35	<.001	15.0	<.001	–22.53	<.001	–32.64	<.001

a Chi-square test for trend: <.001.

Among the total cases, male participants constituted 54.90% (38,519/70,163), while female participants accounted for 45.10% (31,644/70,163), resulting in a male-to-female ratio of 1.22:1. From 2010‐2011 to 2020‐2023, the incidence rate for male increased by 146.72%, whereas the rate for female rose by 169.37% (Table 3).

### Occupational Distribution

**Figure 3.**
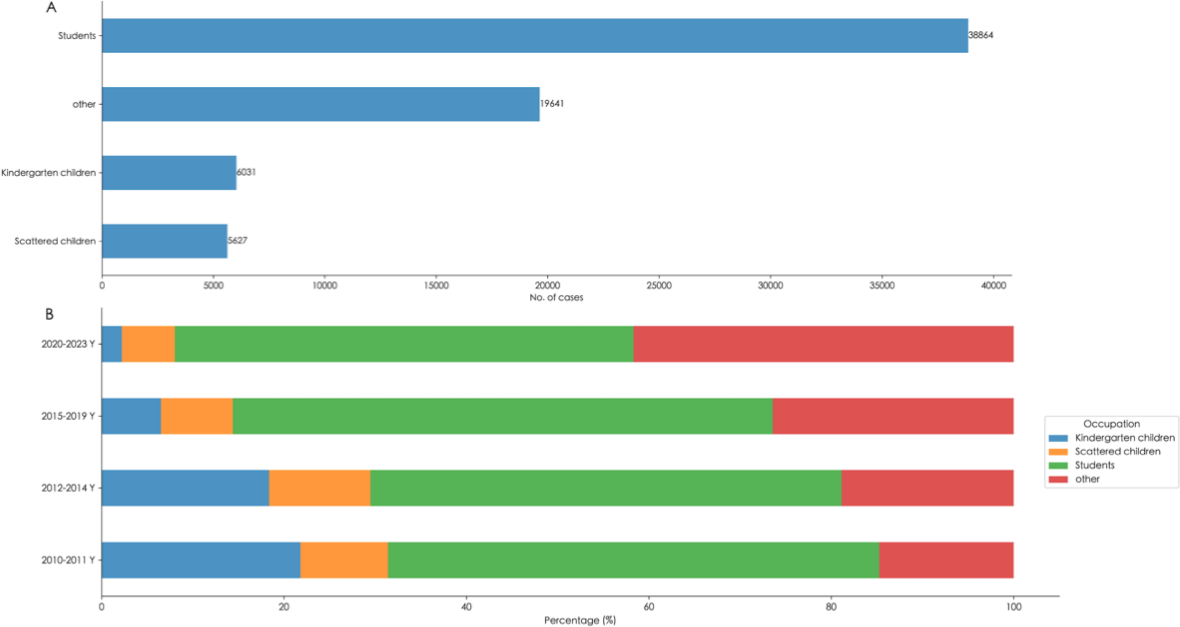
The occupational distribution of varicella by the phases in Ningbo.

Among the cases, there were 38,864 students (comprising 55.39%), 6031 children in childcare (representing 8.60%), 5627 children in disparate circumstances (accounting for 8.02%), and 19,641 instances classified as other nonchildren or students, which collectively constituted 28%. Figure 3 shows the distribution of cases by occupational classification. The proportion of children in childcare significantly declined (*P*<.001), from 21.80% in 2010‐2011 to 2.21% in 2020‐2023. Similarly, the percentage of children in disparate circumstances decreased from 9.61% in 2010‐2011 to 5.82% in 2023 (*P*<.001). In contrast, the proportion of students remained relatively stable, with percentages of 53.84%, 51.67%, 59.19%, and 50.30% during the periods 2010‐2011, 2012‐2013, 2014‐2019, and 2020‐2023, respectively. Conversely, the proportion of other nonchildren or students increased significantly, from 14.75% in 2010‐2011 to 41.67% in 2023 (*P*<.001).

### The Incidence and Number of Varicella Breakthrough Cases

**Figure 4.**
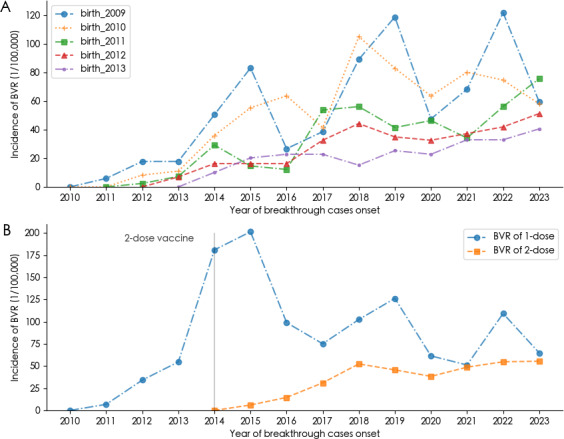
The incidence and number of varicella breakthrough cases in Ningbo. (A) The annual incidence of breakthrough cases by birth cohorts. (B) The annual incidence of breakthrough cases by 1-dose and 2-dose varicella vaccination.

Figure 4A presents the annual incidence of breakthrough varicella infection among different birth cohorts from 2009 to 2013, spanning the years 2010 to 2023. The mean annual breakthrough varicella incidence rate has shown a downward trend in cohorts with more recent birth years compared to those with earlier birth years. Specifically, the breakthrough varicella incidence rate was 53.16 per 100,000 individuals (range, 29.66‐80.07 per 100,000) in 2009, 48.52 per 100,000 (range, 27.62‐71.80 per 100,000) in 2010, 30.64 per 100,000 (range, 14.63‐48.76 per 100,000) in 2011, 23.60 per 100,000 (range, 9.31‐39.56 per 100,000) in 2012, and 17.53 per 100,000 (range, 5.06‐32.91 per 100,000) in 2013.

The average annual incidence of breakthrough cases following a single dose of the varicella vaccine was 83.40 per 100,000 (range, 51.21‐119.50 per 100,000), significantly higher than the incidence after two doses, which was 24.80 per 100,000 (range, 17.67‐32.90 per 100,000). Notably, after the implementation of the 2-dose immunization protocol, the incidence rate of breakthrough cases following the first dose showed a downward trend (Figure 4B).

The median time from vaccination to the occurrence of breakthrough cases was 27 (IQR 17.50‐48) months. For individuals who received a single dose, the median interval was 23 (IQR 17.25‐31.50) months, whereas for those who received two doses, it was 46 (IQR 18‐66) months.

### Impact of NPIs

**Figure 5.**
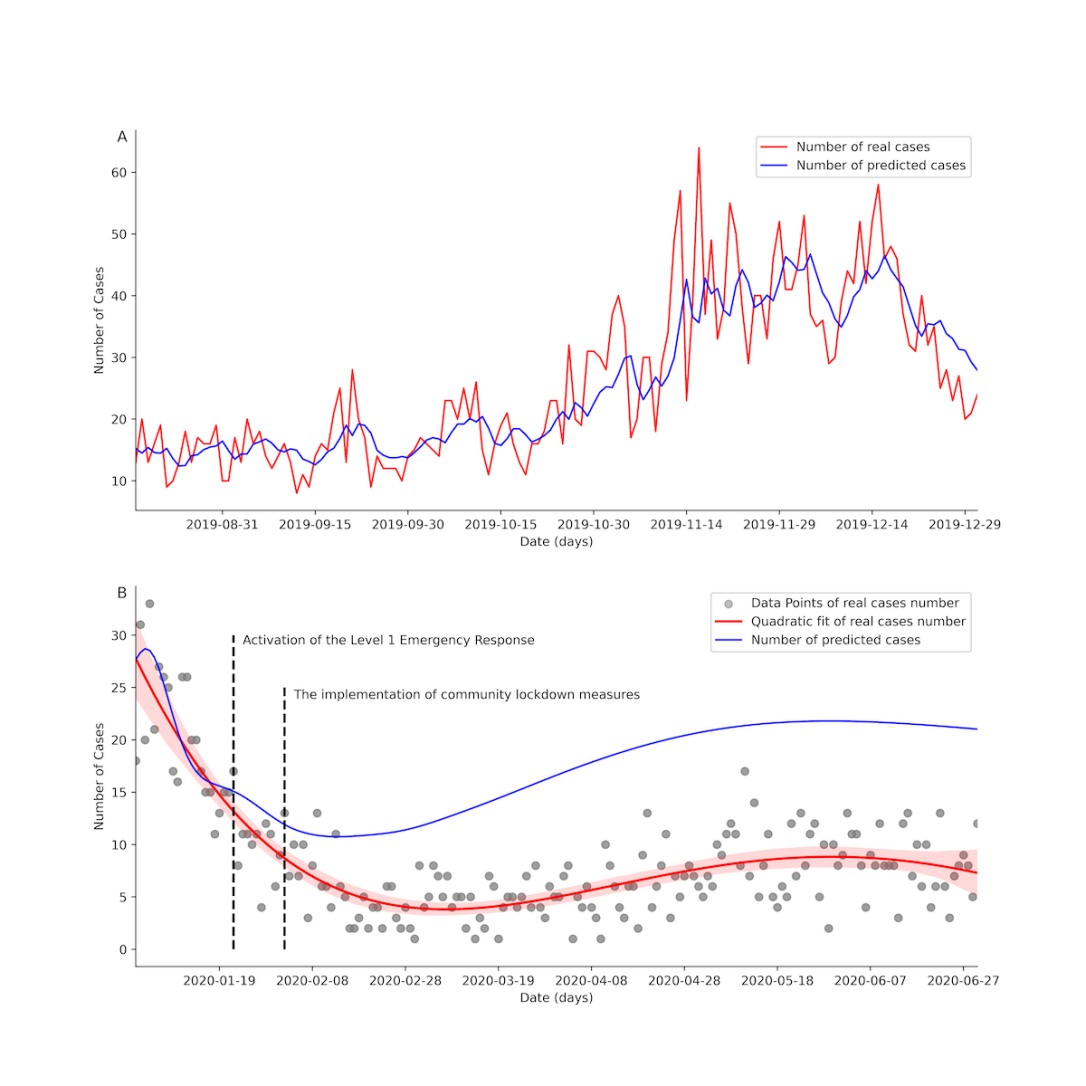
Using recurrent neural network to analyze the impact of nonpharmacological interventions on varicella incidence. (A) The comparison between the actual and predicted case numbers in the validation dataset. (B) The comparison of the predicted and actual varicella case numbers from January 1 to June 30, 2020.

To further analyze the impact of NPIs on the incidence of varicella, we constructed a recurrent neural network model for deep learning. This model features a three-layer neural network architecture with a total of 469,633 trainable parameters. Figure 5A illustrates the comparison between the actual and predicted case numbers in the validation dataset, demonstrating the model’s effectiveness and accuracy in identifying varicella incidence trends. The model achieved a mean squared error of 49.96, a root mean squared error of 7.07, and a mean absolute error of 5.36. Figure 5B presents a comparison of the predicted and actual varicella case numbers from January 1 to June 30, 2020. The results indicate that after the implementation of the Level 1 Emergency Response on January 22, the predicted case numbers gradually exceeded the actual case numbers. Following the implementation of community lockdown measures on February 2, the discrepancy between predicted and actual case numbers significantly increased.

## Discussion

### Principal Findings

In China, varicella is not a legally regulated infectious disease, and the varicella vaccine, as the most effective control measure, is administered voluntarily and at personal expense. Varicella monitoring sites were established to conduct proactive monitoring in Ningbo City. Subsequently, health authorities required web-based direct reporting according to the requirements for reporting Class C infectious diseases. Compared to other studies based largely on voluntary case reporting [14-16], this method can more accurately reflect epidemiological characteristics. This study examined the epidemiological characteristics of varicella from 2010 to 2023, revealing significant changes. The administration of two doses of the varicella vaccine and NPIs had a substantial impact on the epidemiological characteristics.

Previous studies have consistently demonstrated a decline in varicella incidence following the introduction of the varicella vaccine [17-19]. However, our research presents a contrasting trend, revealing an overall increase in varicella incidence from 2010 to 2019. This observation, mirrored by the rising incidence reported in Anhui province from 2012 to 2019 [15], suggests that vaccine impact can be complex and influenced by local epidemiological factors. While our data show a 15% increase in varicella incidence following the implementation of a 2-dose immunization strategy, this overall trend masks important age-specific effects. Notably, we observed a significant decrease in varicella incidence among children younger than 9 years of age, with the most substantial decline (59.54%) in the 1 to 4 age group. This finding aligns with reports from Shi et al [17], who documented a similar reduction in incidence among children aged 0‐4 years in Minhang District, Shanghai, and supports the efficacy of the vaccine in this young age group. However, the contrasting trend of increasing incidence among individuals aged 10 years and older warrants further investigation. This divergence from the findings of Marin et al [18], who reported declines across all groups, highlights the potential for waning immunity or incomplete vaccine coverage in older populations. Shi et al’s [17] report of a 54.09% reduction in incidence among children aged 5‐19 years further underscores the age-specific nature of vaccine effectiveness. Several factors may explain these discrepancies. The timing of 2-dose vaccination programs differs considerably between regions. The United States implemented a routine 2-dose program in 2007, with the second dose administered relatively early at ages 4‐6 years, while this study area implemented the program in 2014. Shanghai’s program, beginning in 2017 and integrated into the local immunization plan by August 2018 with over 80% coverage for the second dose [17], demonstrates the importance of both program timing and achieving high vaccination rates. Our continued observation of increasing incidence among those aged 10‐19 years suggests that lower overall varicella vaccination rates in this cohort may be a key driver.

The optimal timing of the second dose also remains a point of discussion. While the United States and Shanghai recommend it at age 4, Ningbo City administers it at age 3. Sero-epidemiological studies offer conflicting insights: Sun et al [20] suggest a 3-year interval between doses maximizes antibody concentration, while Zhang et al [21] indicate higher seropositivity rates with earlier administration (ages 3‐6). These findings emphasize the need for further research to refine immunization strategies and tailor them to local epidemiological contexts. Ultimately, understanding these nuanced interactions between vaccination programs, age-specific immunity, and population-level factors is crucial for optimizing varicella control and preventing future outbreaks.

During the COVID-19 pandemic, NPIs have demonstrated significant efficacy in controlling varicella outbreaks [22,23]. Originally intended to contain COVID-19, these interventions inadvertently reduced the incidence of other respiratory infectious diseases, such as varicella, due to similar transmission pathways [23]. Measures such as social distancing, mask-wearing, and enhanced hygiene practices led to a notable decline in varicella cases, underscoring their effectiveness in managing respiratory infections. In Xi’an, China, the incidence of varicella decreased by 43.18% in 2020 compared to 2019, attributed to the implementation of COVID-19 NPIs [14]. A European study also suggests that these infection control measures during the pandemic may have curtailed the spread of varicella [24]. Research by Wang et al [25] indicated that the actual number of varicella cases was 93.37% lower than expected, as predicted by the autoregressive integrated moving average model, highlighting a significant reduction in varicella activity due to COVID-19 NPIs. This study revealed that the incidence of chickenpox decreased by an average of 32.64% during the COVID-19 pandemic. Among individuals aged 15 years and older, the rate of increase in chickenpox cases continues to rise; however, this rate is significantly lower compared to the trends observed between 2014 and 2019. Additionally, predictions generated using the long short-term memory (LSTM) model demonstrated that prior to the implementation of COVID-19 prevention and control measures, the predicted number of chickenpox cases closely aligned with the actual reported cases. Following the adoption of these measures, the predicted case numbers consistently exceeded the actual reported cases. Notably, after the introduction of community closure management, the discrepancy between predicted and actual case numbers widened further. These findings suggest that NPIs implemented to curb the spread of COVID-19 also contributed to the prevention and control of chickenpox. Additionally, between 2020 and 2023, the low incidence of varicella from August to September was not significant. This finding aligns with the results of Sabale et al [24], indicating that infection control measures during the pandemic may have altered the seasonal transmission patterns of varicella and reduced its incidence rate.

This study reveals a notable shift in the age distribution of varicella incidence. While consistent with previous reports demonstrating a significant decline in cases among young children [19,26,27], we observed a gradual increase in varicella among individuals aged 10‐14 years between 2010 and 2019, followed by a subsequent rise in cases among those aged 15‐19 years between 2020 and 2023. This contrasts with findings from studies conducted in largely vaccinated populations [28], which have not reported a similar shift toward older age groups. The 95.5% decrease in incidence among children younger than 10 years of age reported by Moek and Siedler [19] highlights the effectiveness of childhood vaccination programs but also underscores the potential for a displacement of incidence to older, unvaccinated or incompletely vaccinated populations. Our finding of increasing incidence among adults aged 20 years and older further supports this hypothesis. The observed epidemiological pattern, with students representing the primary incidence group and a decline in cases among children in childcare, suggests that the implementation of the 2-dose vaccination strategy for children aged 3 years and older is successfully shifting the primary locus of transmission away from early childhood settings like kindergartens. This pattern is consistent with the concept of “herd immunity” waning in older, unvaccinated cohorts, leading to increased susceptibility and transmission within these groups. Further research is needed to investigate the long-term implications of this age-related shift and to inform targeted vaccination strategies for adolescents and adults.

Our initial observations, supported by extensive literature, indicate that the 2-dose varicella vaccine provides substantial epidemiological protection [8,10,29-31] and generally achieves a high vaccination rate. However, breakthrough cases of varicella continue to occur. Liu et al [32] reported that among 6961 varicella cases in Qingyang, China, 28.4% were breakthrough cases. Similarly, Zhu et al [33] documented an increase in the proportion of breakthrough cases during outbreaks, rising from 21.5% in 2008 to 86.1% in 2014, indicating an upward trend. Our findings also demonstrate an increasing incidence of breakthrough cases over time following vaccination. Notably, the incidence of breakthrough cases with the 2-dose vaccine is lower than with a single dose. Furthermore, the implementation of a 2-dose immunization strategy significantly reduced the incidence of breakthrough cases associated with a single dose. The median interval between breakthrough cases was 23 months after a single dose and 46 months after two doses. This suggests that while breakthrough cases still occur after 2 doses, the 2-dose regimen is more effective than a single dose. It not only extends the duration of immune protection but also plays a crucial role in mass immunization efforts.

While the 2-dose varicella vaccine demonstrably provides substantial epidemiological protection and achieves high vaccination rates [8,10,29-31], recent data highlight the ongoing occurrence of breakthrough cases, a phenomenon increasingly recognized in the postvaccination era. Studies from China such as those by Liu et al [32] (28.4% breakthrough rate among 6961 cases) and Zhu et al [33] (increasing from 21.5% in 2008 to 86.1% in 2014) corroborate a growing trend of breakthrough infections, potentially linked to factors such as waning immunity or the emergence of vaccine-escape viral strains. Our findings align with this trend, demonstrating an increasing incidence of breakthrough cases over time following vaccination. Importantly, our data confirm the superior protection offered by the 2-dose regimen compared to a single dose, a finding consistent with established immunological principles regarding vaccine-induced immunity and memory cell development [34]. The significant reduction in breakthrough cases associated with the 2-dose strategy underscores its critical role in sustaining population immunity. The observed median interval of 23 months postsingle dose versus 46 months posttwo doses suggests that the second dose not only enhances initial protection but also extends the duration of immunity, a key consideration for long-term public health strategies. Further research is needed to investigate the factors contributing to breakthrough infections, including viral genetics, host immune responses, and the potential need for booster doses to maintain robust protection.

### Limitations

This study has certain limitations. First, the identification of chickenpox cases in Ningbo primarily relies on clinical assessments. Cases with atypical presentations require evaluation in conjunction with epidemiological histories. However, when cases lack both characteristic clinical features and relevant epidemiological context, accurate identification becomes challenging. As vaccination coverage increases, breakthrough chickenpox cases often present with mild symptoms. Without comprehensive epidemiological investigations, such cases are likely to be overlooked. Furthermore, since 2012, chickenpox surveillance in Ningbo has predominantly relied on passive monitoring. This approach depends on health care providers voluntarily reporting diseases, symptoms, or health events, and it involves compiling data from existing medical records and self-reports from the public. Passive surveillance may lead to diagnostic inaccuracies, such as misclassifying nonchickenpox cases as chickenpox [35]. Additionally, it may result in underreporting, thereby underestimating the true incidence rate. The shift from active to passive monitoring introduces potential biases, particularly in terms of the completeness and representativeness of reports, which can obscure temporal trends and geographic distribution patterns in the data. Active monitoring, while more comprehensive, requires significant labor and time resources, making its consistent implementation across the city challenging. As a result, passive monitoring is widely adopted as a cost-effective alternative for tracking the epidemiological characteristics of infectious diseases over extended periods. To address the limitations of passive monitoring, implementing quality improvement measures is essential. For example, since 2012, Ningbo has integrated chickenpox case reporting into the management framework for Class C statutory infectious diseases. Disease control agencies at various levels are required to conduct regular audits to identify underreporting and provide feedback to improve surveillance accuracy. Based on monitoring outcomes, the system has not indicated significant underreporting of chickenpox cases. Third, LSTMs have demonstrated clear advantages in predicting long-term trends, but they remain unable to accurately model the overall spread of chickenpox during the COVID-19 pandemic. Consequently, the LSTM method is insufficient for evaluating the long-term impact of NPIs on chickenpox incidence when vaccine effects are excluded.

### Conclusions

Through extensive longitudinal surveillance of varicella, we have identified significant changes in its epidemiological characteristics. These include the elimination of the seasonal incidence trough, a shift in the age of onset, and an increasing trend in adult morbidity. These findings highlight the critical need for ongoing epidemiological monitoring and underscore the importance of adapting vaccination strategies to address evolving epidemiological patterns, particularly in the context of postpandemic public health measures. Our investigation further revealed that the incidence of breakthrough cases was markedly lower among individuals who received the 2-dose varicella vaccination regimen compared to those who received only 1 dose. This emphasizes the importance of completing the full vaccination series to achieve higher levels of individual protection and herd immunity. Additionally, the application of machine learning algorithms has demonstrated significant potential in predicting chickenpox incidence. These algorithms enable more effective analysis of the impact of NPIs on disease incidence, providing evidence that NPIs have substantially reduced the burden of chickenpox. The growing use of machine learning in disease surveillance allows researchers to identify previously undetectable patterns and trends, improve predictive accuracy, and facilitate timely interventions. This, in turn, supports the development of more effective disease control measures. In summary, continuous epidemiological surveillance remains essential. We recommend enhanced monitoring of varicella in adults, increased vaccine coverage among individuals aged 15 years and older, and the implementation of 2-dose vaccination programs in high-risk settings such as high schools and universities. For individuals who have received only 1 dose, it is strongly advised to complete the second dose promptly to ensure optimal protection.

## Abbreviations

CDC: Center for Disease Prevention and Control
CISDCP: China Information System for Disease Control and Prevention
LSTM: long short-term memory
NPI: nonpharmaceutical intervention
